# High variation among clinical studies in the assessment of physical function after knee replacement: a systematic review

**DOI:** 10.1007/s00167-023-07375-2

**Published:** 2023-03-13

**Authors:** Marco Adriani, Roland Becker, Giuseppe Milano, Krzysztof Lachowski, Robert Prill

**Affiliations:** 1 Department of Medical and Surgical Specialties, Radiological Sciences, and Public Health, University of Brescia, Brescia, Italy; 2 Center of Orthopaedics and Traumatology, University Hospital Brandenburg/Havel, Brandenburg Medical School Theodor Fontane, Brandenburg a.d.H., Germany; 3 Faculty of Health Sciences Brandenburg, Brandenburg Medical School Theodor Fontane, Brandenburg a.d.H., Germany; 4 Department of Bone and Joint Surgery, Spedali Civili, Brescia, Italy

**Keywords:** Knee arthroplasty, Knee replacement, Outcomes, Domains, Physical function

## Abstract

**Purpose:**

The purpose of this study was to summarise the current use of outcome measures for the assessment of physical function after knee joint replacement.

**Methods:**

A systematic approach following the PRISMA guidelines was used. Literature search was performed on MEDLINE database via PubMed and on Epistemonikos. Clinical trials (level of evidence I-II) on knee joint replacement reporting data on the ‘physical function’ domain published between January 2017 and June 2022 were included. Descriptive statistics were used to summarise the evidence.

**Results:**

In the 181 articles that met the inclusion criteria, 49 different outcome measurements were used to evaluate clinical outcomes after knee joint replacement. The most frequently adopted patient-reported outcome measures (PROMs) were the Knee Society Score (KSS) (78 studies; 43.1%), the Western Ontario and McMaster Universities (WOMAC) Arthritis Index (62 studies; 34.3%), the Oxford Knee Score (OKS) (51 studies; 28.2%) and the Knee Injury and Osteoarthritis Outcome Score (KOOS) (36 studies; 20%). The most frequently used performance-based outcome measures (PBOMs) were the Timed-Up-and-Go (TUG) test (30 studies; 16.6%) and the 6-min-walk test (6MWT) (21 studies; 11.6%). Among impairment-based outcome measures (IBOMs), range of motion (ROM) was the most used (74 studies; 40.9%).

**Conclusion:**

There is considerable variation among clinical studies regarding the assessment of the physical function of patients after knee joint replacement. PROMs were found to be the most commonly adopted outcome measures; however, no single PROM was used in more than half of the papers analysed.

**Level of evidence:**

Level II, systematic review of level I-II studies.

**Electronic supplementary material:**

The online version contains supplementary material available at 10.1007/s00167-023-07375-2.

## Introduction

The available literature on joint replacement is continuously growing; however, there is heterogeneity in the outcome measurement instruments used in clinical trials [20]. In 2017, the Outcome Measures in Rheumatology Clinical Trials (OMERACT) total joint replacement (TJR) special interest group published a core domain set for assessing outcomes after TJR of the hip and knee using an onion model [16, 21]. The ‘physical function’ domain was included in the inner circle of the model, meaning that it is a core domain that must be included in every trial. However, the OMERACT working group, like others researchers, continued to debate how to measure and define a physical function [22].

Outcome measures used to assess physical function after knee joint replacement can be divided into three main categories [17]: patient-reported outcome measures (PROMs), performance-based outcome measures (PBOMs) and impairment-based outcome measures (IBOMs), based on findings of impaired joint mobility, muscle performance, and range of motion (ROM). Each of these categories is used to assess a different construct of physical function and the results are complementary. Specifically, PROMs provide insights into the effectiveness of care from the patient’s perspective and therefore eliminate biases related to the clinician’s evaluation [6, 25]. Conversely, PBOMs and IBOMs are objective measurements that do not rely on the patient’s memory, self-assessment or sense of judgement [6]. Notably, unlike PBOMs, IBOMs address only a single aspect of knee function and do not provide a comprehensive overview of the domain. Furthermore, many PROMs have been described in recent years to assess physical function after knee replacement, whereas PBOMs and IBOMs are limited in number and scope [3].

The purpose of the present study was to summarise the current use of outcome measures for the assessment of physical function after knee joint replacement. The hypothesis of the study was that there is a large difference across studies in the use of outcome measurement instruments for the assessment of physical function after knee joint replacement.

## Methods

A systematic approach that followed the Knee Surgery, Sports Traumatology, Arthroscopy (KSSTA) authors’ guidelines for systematic reviews was used [15]. The updated Preferred Reporting Items for Systematic Reviews and Meta-Analysis (PRISMA) 2020 guidelines for systematic reviews were followed [12]. The study was preregistered with the Open Science Framework (https://doi.org/10.17605/OSF.IO/T6K8Q). To identify potentially relevant studies for inclusion, a literature search was performed in the MEDLINE database via PubMed and Epistemonikos using the following keywords: ‘knee arthroplasty’, ‘knee replacement’, ‘total knee’, ‘partial knee’ and ‘unicompartmental knee’. Studies published between January 2017 (the year the OMERACT consensus was published) and June 2022 were included in the systematic review. The search strategy is presented in Additional file 1.

The inclusion criteria were as follows: (a) a randomised controlled trial (level of evidence I–II), (b) pertaining to total or unicompartmental knee joint replacement, (c) reporting on functional outcomes, (d) published in the last 5 years and (e) in English, Italian or German language.

The following were the exclusion criteria: (a) a case series, case–control study, cohort study, case report, review or an expert opinion, (b) no involvement of human beings or a cadaveric study, (c) a radiographic or an anaesthesiology study and (d) no functional outcome data at a minimum of 3 months of follow-up. Also, studies on the usage of tranexamic acid were excluded, as their main outcomes are not related to physical function but to blood loss and related areas.

One author (MA) conducted the search on June 14th, 2022. Two authors (MA and PR) screened the titles and abstracts for the inclusion criteria and solved disagreement by discussion. The authors compiled a list of articles after the application of the inclusion and exclusion criteria. No further articles were excluded after the full-text screening. Due to the nature of this review, a critical appraisal was not feasible. Two reviewers collected data from the included studies independently, without the usage of automation tools. Disagreements in data extraction were double-checked and the authors defined the results together.

During the initial data review, the following information was collected from each study: title, authors, year of publication, study design, outcomes evaluated, and the main topic of the trial. FOr this purpose, the studies were assigned to three different categories according to the main investigated topic: (a) surgery-related studies (exploring implant design, implant fixation, approach, referencing and instrumentation, such as robotics and similar); (b) post-operative rehabilitation-related studies; and (c) studies related to other areas. Discrepancies that arose between the authors about the main topic of a study were resolved by discussion. The outcome measures used to assess the function domain were then divided into three categories: PROMs, PBOMs and IBOMs. Due to the nature of this review, critical appraisal was not feasible. Descriptive statistics were used to summarise the evidence.

## Results

**Fig. 1:**
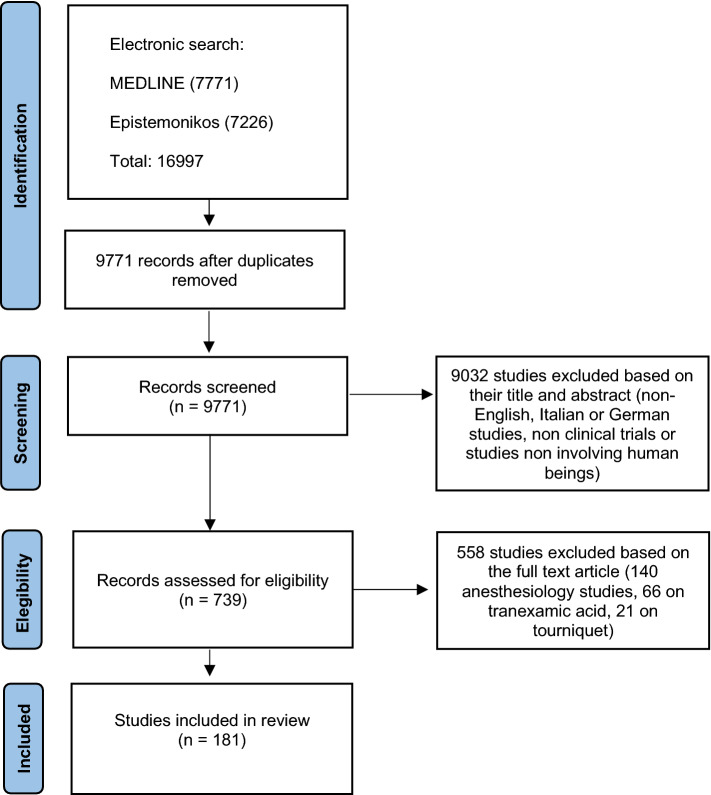
PRISMA flow diagram

After the removal of duplicates, the literature search yielded 9771 studies (Fig. 1). Of these, 181 articles met the inclusion criteria (Additional file 2). For the assessment of functional outcomes after knee joint replacement, 26 different PROMs, 16 PBOMs and 7 IBOMs were used in the included articles.

**Fig. 2:**
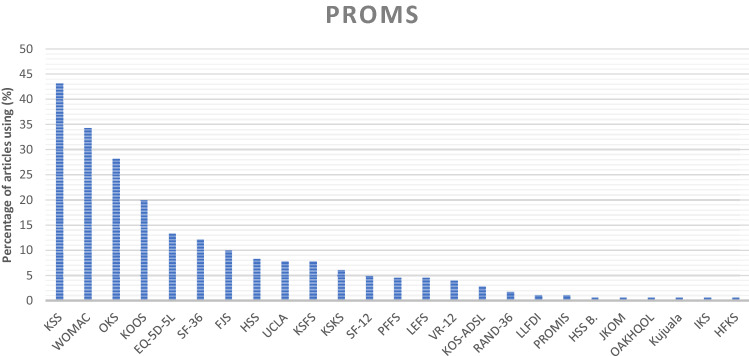
Distribution of patient-reported outcome measures among the articles included in the review. *KSS = The 2011 Knee Society Scoring System, *EQ-5D-5L* = EuroQol-5 Dimension 5-level, SF-36 = Short form health survey-36, *FJS *= forgotten joint score, *HSS* = Hospital for special surgery; UCLA = University of California Los Angeles activity score, *KSFS* = Knee society function score; *KSKS *= Knee society knee score; *SF-12* = MOS short form-12, *PFFS* = Patellofemoral Feller Score; *LEFS* = lower extremity functional scale; *VR-1*2 = Veterans RAND 12-Item health survey; KOS-*ADSL* = activities of the daily living scale of the knee outcome survey, *LLFDI* = late-life function and disability index, *PROMIS* = patient-reported outcomes measurement information system, *HSS B* = HSS Baldini, *JKOM* = Japanese Knee Osteoarthritis Measure, *OAKHQOL* = Osteoarthritis Knee and hip quality of life, *IKS* = Insall knee score, *HFKS* = High flexion knee score

Overall, 173 studies (95.6%) used at least one PROM to describe physical function after knee joint replacement. The most frequently used were the 2011 Knee Society Score (KSS) (78 studies), the Western Ontario and McMaster Universities Arthritis Index (WOMAC) (62 studies), the Oxford Knee Score (OKS) (51 studies) and the Knee Injury and Osteoarthritis Outcome Score (KOOS) (36 studies). Other PROMs were used in less than 15% of the studies (Fig. 2).

**Fig. 3:**
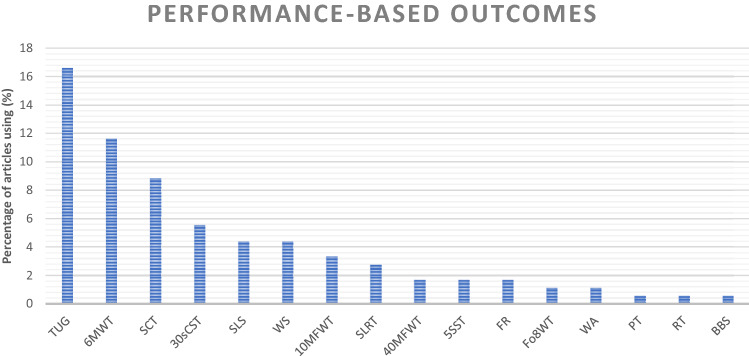
Distribution of performance-based outcome measures among the articles included in the review *TUG = Timed up and go test, *6MWT *= 6-Minute Walk Test, *SCT* = Stair climb test, *30sCST* = 30 s chair-stand test, *SLS* = single leg stance, *WS* = walking speed, *10MFWT* = 10 m Fast Walk Test, SLRT = Straight Leg Raising Time, *40MFWT* = 40 m Fast Walk Test, *5SST *= 5-time sit to stand test, *FR* = Forward reach, *Fo8WT* = Figure of 8 walk test, *WA* = walking ability, *PT *= Proprioception tests, *RT* = Roomberg test, BBS = berg balance test.

Fifty-seven studies (31.5%) used at least one PBOM to analyse physical function. The most frequently used were the Timed-Up-and-Go test (TUG) (30 studies) and the six-minute-walk test (6MWT) (21 studies). Other PBOMs were used in less than 10% of the studies** (**Fig. 3).

**Fig. 4:**
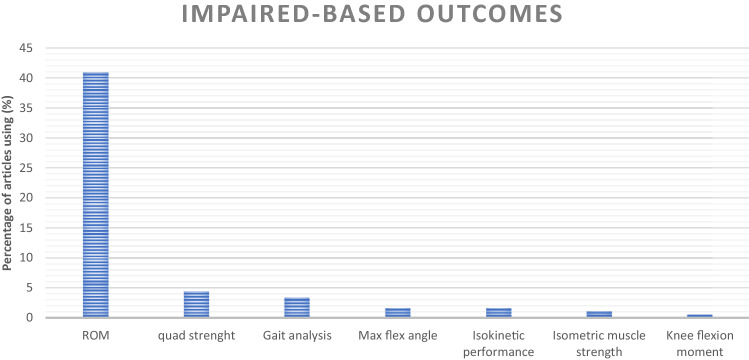
Distribution of impairment-based outcome measures among the articles included in the review

Regarding IBOMs, ROM was used in 74 studies, whereas all the other IBOMs were used in less than 5% of the studies (Fig. 4).

Some studies used more than one category of functional outcome, such as one PROM and one PBOM or IBOM or all three types of measure. Overall, 51 articles (28.2%) used one PROM and one PBOM, 79 articles (43.7%) used one PROM and one IBOM and only 31 papers (17.1%) adopted all three types of measure.

By analysing differences in the outcome measurement instruments used to assess physical function according to the main topic of the included studies (Table 1), we observed that post-operative rehabilitation-related studies adopted PBOMs more than surgery-related studies (68.9% and 12.2%, respectively).

**Table 1:** Comparison of outcome measures used per main topic of the trial

	*N*° studies included %	*N*° studies using PROMs %	*N*° studies using PBOMs %	*N*° studies using IBOMs %
Surgery-related studies	115 (63.5)	109 (94.8)	47 (40.9)	14 (12.2)
Post-operative rehabilitation-related studies	45 (24.9)	31 (68.9)	41 (91.1)	25 (55.6)
Others	23 (12.7)	23 (100)	12 (52.2)	12 (52.2)

Minimal clinically important differences (MCID) for functional outcomes was reported in only seven studies (3.9%) [1, 8, 9, 11, 13, 19, 23].

## Discussion

The main finding of this review was the considerable variation in the literature regarding the outcome assessment of patients after knee joint replacement. On 181 level-I or II included that were published over the last five years, 49 different outcome measures were identified, and none of the outcome measurements were used in more than 50% of the studies. These findings are relevant, as having a plethora of outcomes across different studies makes it difficult to summarise data in systematic reviews and meta-analyses with sufficient power to draw any conclusions.

Findings of the present study are in accordance with those of previous systematic reviews published before the OMERACT consensus was released [21], which already highlighted the high variation in the outcome measures used in studies on joint replacement [7, 20]. Differently from those previous systematic reviews [7, 20], in the present study only randomised controlled trials conducted on both total and unicompartmental knee replacement were selected, and only outcome measures related to physical function were evaluated. Nevertheless, a more homogeneous use of outcome measures was expected, at least in high-level studies, after the release of the endorsement by OMERACT, because specific guidelines were provided for authors [21]. However, this was found to not be the case.

PROMs were more frequently used than PBOMs for the assessment of physical function. Specifically, 41% of the included studies adopted only PROMs for the assessment of physical function. Post-operative rehabilitation-related studies adopted PBOMs more than surgery-related studies. This was not surprising, as PBOMs have been studied and applied to a greater extent by physiotherapists than by orthopaedic surgeons [24].

Although PROMs are relevant to patient’s perspective, they are often affected by factors not directly related to the surgical outcome. Some systematic reviews have questioned the reliability of PROMs. Particularly, WOMAC and OKS demonstrated a ceiling effect in patients after total knee joint replacement. This may explain the concern that lower discriminatory power leads to less ability to recognise small improvements within and between patients [4, 10].

A recent study investigated the reliability and agreement of four PBOMs and one functional test in patients undergoing total knee replacement and correlated them with two PROMs [14]. Interestingly, all the PBOMs showed excellent reliability; however, there was a lack of clinically relevant correlation between symmetry-related PBOMs and PROMs. This highlights the importance of including PBOMs that depict a different aspect of physical function after knee replacement. Therefore, future studies should not solely report functional outcomes based on PROMs. It is advisable to include at least one PBOMs in each patient’s clinical evaluation.

PBOMs assess the performance of simple and specific tasks in the outpatient setting. Their main limitation is the presence of the ‘practical effect’, meaning that performance will improve with multiple tests [5]. Hence, testing requires appropriate space and time, and as a result, feasibility is limited. This might explain why IBOMs based on a single specific physical attribute, like ROM (used in 40.9% of the included studies), are frequently adopted despite their lack of accuracy.

There is not enough evidence on the superiority of one outcome measurement over another in depicting physical function after knee joint replacement. Future studies should focus on comparing these instruments to let the development of a core outcome set possible. Nevertheless, researchers should adopt the outcome measures with the most extensively validated measurement instruments to decrease the uncertainty of measurements and discrepancies in results. KOOS, WOMAC and OKS are the only PROMs that have been extensively validated in patients undergoing knee joint replacement, whereas TUG and 6MWT proved to be the most extensively validated PBOMs [18].

In addition to appropriate patient assessment, clinical relevance of statistically significant differences in outcome measures should be discussed more critically. Analysis of the MCID is recommended, which reflects changes in the clinical intervention that are meaningful for the patient [2]. However, among the studies included in the present study, only a few included analysis of the MCID [1, 8, 9, 11, 13, 19, 23].

This review has some limitations. First, it was decided to include only articles published in the last five years and available via PubMed or Epistemonikos. Furthermore, only randomised controlled trials were included and thus some of the functional outcome measures used in prospective observational and clinical cross-sectional studies might have been missed. This could result in an incomplete overview of the measures adopted in the literature. However, the objective was not to summarise all data available in the literature on knee replacement but to increase awareness in terms of the quality of assessment of physical function.

## Conclusions

The findings of this review showed that there is considerable variation among clinical trials on knee joint replacement in terms of outcome measures used to evaluate the domain of physical function. PROMs are the most widely adopted outcomes; however, none of them was used in more than half of the studies. In addition, it was found that a small percentage of studies use PBOMs, except studies focused on post-operative rehabilitation.

## Supplementary Information

Below is the link to the electronic supplementary material.

Supplementary file 1 (PDF 8 KB) Search strategy. This file provides the search strategy adopted for the review.

Supplementary file 2 (PDF 194 KB) Included articles. This file contains the references of all the included studies.

## Abbreviations

OMERACT: Outcome measures in rheumatology clinical trials
TJR: Total joint replacement
PROMs: Patient-reported outcome measures
PBOMs: Performance-based outcome measures
IBOMs: Impairment-based outcome measures
ROM: Range of motion
KOOS: Knee injury and osteoarthritis outcome score
WOMAC: Western Ontario and Mcmaster Universities Arthritis Index
OKS: Oxford knee score
MCID: Minimal clinically important differences
