# Supplementary material for: High variation among clinical studies in the assessment of physical function after knee replacement: a systematic review
Source: Knee Surg Sports Traumatol Arthrosc. 2023 Mar 13;31(9):3854–60. doi: 10.1007/s00167-023-07375-2 (PMC10435639; doi:10.1007/s00167-023-07375-2)
Supplement: Supplementary file 1 — (PDF 8 KB) Search strategy. This file provides the search strategy adopted for the review. [file 167_2023_7375_MOESM1_ESM.pdf]

Additional file 1: Search strategy

total knee

partial knee

knee replacement

unicompartmental knee

knee arthropl*

knee prosthes*

1 or 2or 3 or 4 or 5 or 6

7 AND (2017:2022 [pdat])
