# Supplementary material for: High variation among clinical studies in the assessment of physical function after knee replacement: a systematic review
Source: Knee Surg Sports Traumatol Arthrosc. 2023 Mar 13;31(9):3854–60. doi: 10.1007/s00167-023-07375-2 (PMC10435639; doi:10.1007/s00167-023-07375-2)
Supplement: Supplementary file 2 — (PDF 194 KB) Included articles. This file contains the references of all the included studies. [file 167_2023_7375_MOESM2_ESM.pdf]

Additional file 2: Included articles

1. Abane L, Zaoui A, Anract P, Lefevre N, Herman S, Hamadouche M (2018) Can a Single-Use and Patient-Specific Instrumentation Be Reliably Used in Primary Total Knee Arthroplasty? A Multicenter Controlled Study. J Arthroplasty 33:2111–2118
2. Abdel MP, Tibbo ME, Stuart MJ, Trousdale RT, Hanssen AD, Pagnano MW (2018) A randomized controlled trial of fixed- versus mobile-bearing total knee arthroplasty: a follow-up at a mean of ten years. Bone Joint J 100-B:925–929
3. Akti S, Karakus D, Sezgin EA, Cankaya D (2021) No differences in clinical outcomes or isokinetic performance between cruciate-substituting ultra-congruent and posterior stabilized total knee arthroplasties: a randomized controlled trial. Knee Surg Sports Traumatol Arthrosc 29:3443–3449
4. Ali SF, Gharaibeh MA, Wood JA, Chen DB, MacDessi SJ (2021) No difference in clinical outcomes between portable navigation and conventional instrumentation in total knee arthroplasty: A randomised trial. ANZ J Surg 91:1914–1918
5. Alvand A, Khan T, Jenkins C, Rees JL, Jackson WF, Dodd CAF, et al. (2018) The impact of patient-specific instrumentation on unicompartmental knee arthroplasty: a prospective randomised controlled study. Knee Surg Sports Traumatol Arthrosc 26:1662–1670
6. Amaro JT, Novaretti JV, Astur DC, Cavalcante ELB, Rodrigues Junior AG, Debieux P, et al. (2020) Higher Axial Tibiofemoral Rotation and Functional Outcomes with Mobile-Bearing Compared with Fixed-Bearing Total Knee Arthroplasty at 1- but Not at 2-Year Follow-Up-A Randomized Clinical Trial. J Knee Surg 33:474–480
7. Andrade MAP, Monte LFR, Lacerda GC, Dourado TR, Lei P, Abreu-E-Silva GM (2022) Are cementation quality and clinical outcomes affected by the use of tourniquet in primary total knee arthroplasty? Arch Orthop Trauma Surg 142:845–850
8. Aslam MA, Sabir AB, Tiwari V, Abbas S, Tiwari A, Singh P (2017) Approach to Total Knee Replacement: A Randomized Double Blind Study between Medial Parapatellar and Midvastus Approach in the Early Postoperative Period in Asian Population. J Knee Surg 30:793–797
9. Bäcker HC, Wu CH, Schulz MRG, Weber-Spickschen TS, Perka C, Hardt S (2021) App-based rehabilitation program after total knee arthroplasty: a randomized controlled trial. Arch Orthop Trauma Surg 141:1575–1582
10. Bade MJ, Struessel T, Dayton M, Foran J, Kim RH, Miner T, et al. (2017) Early High-Intensity Versus Low-Intensity Rehabilitation After Total Knee Arthroplasty: A Randomized Controlled Trial. Arthritis Care Res (Hoboken) 69:1360–1368
11. Banger MS, Doonan J, Jones BG, MacLean AD, Rowe PJ, Blyth MJG (2022) Are there functional biomechanical differences in robotic arm-assisted bi-unicompartmental knee arthroplasty compared with conventional total knee arthroplasty? A prospective, randomized controlled trial. Bone Joint J104-B:433–443
12. Banger MS, Johnston WD, Razii N, Doonan J, Rowe PJ, Jones BG, et al. (2020) Robotic arm-assisted bi-unicompartmental knee arthroplasty maintains natural knee joint anatomy compared with total knee arthroplasty: a prospective randomized controlled trial. Bone Joint J 102-B:1511–1518
13. Barker KL, Room J, Knight R, Dutton S, Toye F, Leal J, et al. (2021) Home-based rehabilitation programme compared with traditional physiotherapy for patients at risk of

poor outcome after knee arthroplasty: the CORKA randomised controlled trial. BMJ Open 11:e052598
14. Barker KL, Room J, Knight R, Dutton SJ, Toye F, Leal J, et al. (2020) Outpatient physiotherapy versus home-based rehabilitation for patients at risk of poor outcomes after knee arthroplasty: CORKA RCT. Health Technol Assess 24:1–116
15. Batra S, Malhotra R, Kumar V, Srivastava DN, Backstein D, Pandit H (2021) Superior patient satisfaction in medial pivot as compared to posterior stabilized total knee arthroplasty: a prospective randomized study. Knee Surg Sports Traumatol Arthrosc 29:3633–3640
16. Beard DJ, Davies LJ, Cook JA, MacLennan G, Price A, Kent S, et al. (2020) Total versus partial knee replacement in patients with medial compartment knee osteoarthritis: the TOPKAT RCT. Health Technol Assess 24:1–98
17. Behrend H, Lengnick H, Zdravkovic V, Ladurner A, Rudin D, Erschbamer M, et al. (2019) Vitamin C demand is increased after total knee arthroplasty: a double-blind placebo-controlled-randomized study. Knee Surg Sports Traumatol Arthrosc 27:1182–1188
18. Bernal-Fortich LD, Aguilar CA, Rivera-Villa AH, Galindo-Avalos J, Aguilera-Martínez P, Torres-González R, et al. (2018) A prospective randomized trial of total synovectomy versus limited synovectomy in primary total knee arthroplasty: evaluation of bleeding, postoperative pain, and quality of life with SF-12 v2. Eur J Orthop Surg Traumatol 28:701–706
19. Beyer F, Pape A, Lützner C, Kirschner S, Lützner J (2021) Similar outcomes in computer-assisted and conventional total knee arthroplasty: ten-year results of a prospective randomized study. BMC Musculoskelet Disord 22:707
20. Blasco J-M, Acosta-Ballester Y, Martínez-Garrido I, García-Molina P, Igual-Camacho C, Roig-Casasús S (2020) The effects of preoperative balance training on balance and functional outcome after total knee replacement: a randomized controlled trial. Clin Rehabil 34:182–193
21. van den Boom LGH, Brouwer RW, van den Akker-Scheek I, Reininga IHF, de Vries AJ, Bierma-Zeinstra SMA, et al. (2020) No Difference in Recovery of Patient-Reported Outcome and Range of Motion between Cruciate Retaining and Posterior Stabilized Total Knee Arthroplasty: A Double-Blind Randomized Controlled Trial. J Knee Surg 33:1243–1250
22. Breddam Mosegaard S, Rytter S, Madsen F, Odgaard A, Søballe K, Stilling M (2021) Two-year fixation and ten-year clinical outcomes of total knee arthroplasty inserted with normal-curing bone cement and slow-curing bone cement: A randomized controlled trial in 54 patients. Knee 33:110–124
23. Briones-Cantero M, Fernández-de-Las-Peñas C, Lluch-Girbés E, Osuna-Pérez MC, Navarro-Santana MJ, Plaza-Manzano G, et al. (2020) Effects of Adding Motor Imagery to Early Physical Therapy in Patients with Knee Osteoarthritis who Had Received Total Knee Arthroplasty: A Randomized Clinical Trial. Pain Med 21:3548–3555
24. Brown ML, Wendt CS, Seyler TM, Ip EH, Foxworth JL, Lang JE (2019) Gait and Functional Outcomes Between Cruciate-Retaining and Cruciate-Substituting Implants in Total Knee Arthroplasty: A Prospective, Randomized Study. J Surg Orthop Adv 28:215–223

25. Cai L, Gao H, Xu H, Wang Y, Lyu P, Liu Y (2018) Does a Program Based on Cognitive Behavioral Therapy Affect Kinesiophobia in Patients Following Total Knee Arthroplasty? A Randomized, Controlled Trial With a 6-Month Follow-Up. J Arthroplasty 33:704–710
26. Calatayud J, Casaña J, Ezzatvar Y, Jakobsen MD, Sundstrup E, Andersen LL (2017) High-intensity preoperative training improves physical and functional recovery in the early post-operative periods after total knee arthroplasty: a randomized controlled trial. Knee Surg Sports Traumatol Arthrosc 25:2864–2872
27. Chang JS, Kayani B, Moriarty PD, Tahmassebi JE, Haddad FS (2021) A Prospective Randomized Controlled Trial Comparing Medial-Pivot versus Posterior-Stabilized Total Knee Arthroplasty. J Arthroplasty 36:1584-1589.e1
28. Chaudhry A, Goyal VK (2018) Fixed-bearing versus high-flexion RP total knee arthroplasty (TKA): midterm results of a randomized controlled trial. J Orthop Traumatol 19:2
29. Chen W, Sun J-N, Hu Z-H, Zhang Y, Chen X-Y, Feng S (2021) Cognitive behavioral therapy cannot relieve postoperative pain and improve joint function after total knee arthroplasty in patients aged 70 years and older. Aging Clin Exp Res 33:3293–3302
30. Cip J, Obwegeser F, Benesch T, Bach C, Ruckenstuhl P, Martin A (2018) Twelve-Year Follow-Up of Navigated Computer-Assisted Versus Conventional Total Knee Arthroplasty: A Prospective Randomized Comparative Trial. J Arthroplasty 33:1404–1411
31. Clark AN, Hounat A, O'Donnell S, May P, Doonan J, Rowe P, et al. (2021) Electromagnetic Navigated Versus Conventional Total Knee Arthroplasty-A Five-Year Follow-Up of a Single-Blind Randomized Control Trial. J Arthroplasty 36:3451–3455
32. Crawford DA, Duwelius PJ, Sneller MA, Morris MJ, Hurst JM, Berend KR, et al. (2021) 2021 Mark Coventry Award: Use of a smartphone-based care platform after primary partial and total knee arthroplasty: a prospective randomized controlled trial. Bone Joint J 103-B:3–12
33. Debbi EM, Bernfeld B, Herman A, Salai M, Laufer Y, Wolf A (2019) A Biomechanical Foot-Worn Device Improves Total Knee Arthroplasty Outcomes. J Arthroplasty 34:47–55
34. DeJong G, Hsieh CJ, Vita MT, Zeymo A, Boucher HR, Thakkar SC (2020) Innovative Devices Did Not Provide Superior Total Knee Arthroplasty Outcomes in Post-Operative Rehabilitation: Results From a Four-Arm Randomized Clinical Trial. J Arthroplasty 35:2054–2065
35. Deroche E, Batailler C, Swan J, Sappey-Marinier E, Neyret P, Servien E, et al. (2022) No difference between resurfaced and non-resurfaced patellae with a modern prosthesis design: a prospective randomized study of 250 total knee arthroplasties. Knee Surg Sports Traumatol Arthrosc 30:1025–1038
36. Domínguez-Navarro F, Silvestre-Muñoz A, Igual-Camacho C, Díaz-Díaz B, Torrella JV, Rodrigo J, et al. (2021) A randomized controlled trial assessing the effects of preoperative strengthening plus balance training on balance and functional outcome up to 1 year following total knee replacement. Knee Surg Sports Traumatol Arthrosc 29:838–848
37. Dong Y, Li T, Zheng Z, Xiang S, Weng X (2018) Adding Patella Resurfacing After Circumpatellar Electrocautery Did Not Improve the Clinical Outcome in Bilateral Total Knee Arthroplasty in Chinese Population: A Prospective Randomized Study. J Arthroplasty 33:1057–1061

38. Dowsey MM, Brown WA, Cochrane A, Burton PR, Liew D, Choong PF (2022) Effect of Bariatric Surgery on Risk of Complications After Total Knee Arthroplasty: A Randomized Clinical Trial. JAMA Netw Open 5:e226722
39. Dowsey MM, Gould DJ, Spelman T, Pandy MG, Choong PF (2020) A Randomized Controlled Trial Comparing a Medial Stabilized Total Knee Prosthesis to a Cruciate Retaining and Posterior Stabilized Design: A Report of the Clinical and Functional Outcomes Following Total Knee Replacement. J Arthroplasty 35:1583-1590.e2
40. Edelstein AI, Bhatt S, Wright-Chisem J, Sullivan R, Beal M, Manning DW (2020) The Effect of Implant Design on Sagittal Plane Stability: A Randomized Trial of Medial- versus Posterior-Stabilized Total Knee Arthroplasty. J Knee Surg 33:452–458
41. Eichler S, Rabe S, Salzwedel A, Müller S, Stoll J, Tilgner N, et al. (2017) Effectiveness of an interactive telerehabilitation system with home-based exercise training in patients after total hip or knee replacement: study protocol for a multicenter, superiority, no-blinded randomized controlled trial. Trials 18:438
42. Eymir M, Erduran M, Ünver B (2021) Active heel-slide exercise therapy facilitates the functional and proprioceptive enhancement following total knee arthroplasty compared to continuous passive motion. Knee Surg Sports Traumatol Arthrosc 29:3352–3360
43. Feczko PZ, Jutten LM, van Steyn MJ, Deckers P, Emans PJ, Arts JJ (2017) Comparison of fixed and mobile-bearing total knee arthroplasty in terms of patellofemoral pain and function: a prospective, randomised, controlled trial. BMC Musculoskelet Disord 18:279
44. Fillingham YA, Darrith B, Lonner JH, Culvern C, Crizer M, Della Valle CJ (2018) Formal Physical Therapy May Not Be Necessary After Unicompartmental Knee Arthroplasty: A Randomized Clinical Trial. J Arthroplasty 33:S93-S99.e3
45. Fransen BL, Hoozemans MJM, Argelo KDS, Keijser LCM, Burger BJ (2018) Fast-track total knee arthroplasty improved clinical and functional outcome in the first 7 days after surgery: a randomized controlled pilot study with 5-year follow-up. Arch Orthop Trauma Surg 138:1305–1316
46. Fricka KB, McAsey CJ, Sritulanondha S (2019) To Cement or Not? Five-Year Results of a Prospective, Randomized Study Comparing Cemented vs Cementless Total Knee Arthroplasty. J Arthroplasty 34:S183–S187
47. Giannotti S, Sacchetti F, Citarelli C, Bottai V, Bianchi N, Agostini G, et al. (2020) Single-use, patient-specific instrumentation technology in knee arthroplasty: a comparative study between standard instrumentation and PSI efficiency system. Musculoskelet Surg 104:195–200
48. Gilmour A, MacLean AD, Rowe PJ, Banger MS, Donnelly I, Jones BG, et al. (2018) Robotic-Arm-Assisted vs Conventional Unicompartmental Knee Arthroplasty. The 2-Year Clinical Outcomes of a Randomized Controlled Trial. J Arthroplasty 33:S109–S115
49. Goh JKM, Chen JY, Yeo NEM, Liow MHL, Chia S-L, Yeo SJ (2020) Ten year outcomes for the prospective randomised trial comparing unlinked, modular bicompartmental knee arthroplasty and total knee arthroplasty. Knee 27:1914–1922
50. Goicoechea N, Hinarejos P, Torres-Claramunt R, Leal-Blanquet J, Sánchez-Soler J, Monllau JC (2021) Patellar denervation does not reduce post-operative anterior knee pain after primary total knee arthroplasty with patellar resurfacing. Knee Surg Sports Traumatol Arthrosc 29:3346–3351

51. Ha C, Wang B, Li W, Sun K, Wang D, Li Q (2019) Resurfacing versus not-resurfacing the patella in one-stage bilateral total knee arthroplasty: a prospective randomized clinical trial. Int Orthop 43:2519–2527
52. van Hamersveld KT, Marang-van de Mheen PJ, Tsonaka R, Valstar ER, Toksvig-Larsen S (2017) Fixation and clinical outcome of uncemented peri-apatite-coated versus cemented total knee arthroplasty : five-year follow-up of a randomised controlled trial using radiostereometric analysis (RSA). Bone Joint J 99-B:1467–1476
53. Hamilton DF, Beard DJ, Barker KL, Macfarlane GJ, Tuck CE, Stoddart A, et al. (2020) Targeting rehabilitation to improve outcomes after total knee arthroplasty in patients at risk of poor outcomes: randomised controlled trial. BMJ 371:m3576
54. Hamilton DF, Burnett R, Patton JT, MacPherson GJ, Simpson AHRW, Howie CR, et al. (2020) Reduction in patient outcomes but implant-derived preservation of function following total knee arthroplasty: longitudinal follow-up of a randomized controlled trial. Bone Joint J 102-B:434–441
55. Hampton M, Mansoor J, Getty J, Sutton PM (2020) Uncemented tantalum metal components versus cemented tibial components in total knee arthroplasty: 11- to 15-year outcomes of a single-blinded randomized controlled trial. Bone Joint J 102-B:1025–1032
56. Hasan S, Marang-Van De Mheen PJ, Kaptein BL, Nelissen RGHH, Toksvig-Larsen S (2019) All-polyethylene versus metal-backed posterior stabilized total knee arthroplasty: similar 2-year results of a randomized radiostereometric analysis study. Acta Orthop 90:590–595
57. Hauer G, Hörlesberger N, Klim S, Bernhardt GA, Leitner L, Glehr M, et al. (2021) Mid-term results show no significant difference in postoperative clinical outcome, pain and range of motion between a well-established total knee arthroplasty design and its successor: a prospective, randomized, controlled trial. Knee Surg Sports Traumatol Arthrosc 29:827–831
58. Henricson A, Wojtowicz R, Nilsson KG, Crnalic S (2019) Uncemented or cemented femoral components work equally well in total knee arthroplasty. Knee Surg Sports Traumatol Arthrosc 27:1251–1258
59. Hepperger C, Gföller P, Hoser C, Ulmer H, Fischer F, Schobersberger W, et al. (2017) The effects of a 3-month controlled hiking programme on the functional abilities of patients following total knee arthroplasty: a prospective, randomized trial. Knee Surg Sports Traumatol Arthrosc 25:3387–3395
60. Hernigou P, Auregan JC, Dubory A, Flouzat-Lachaniette CH, Chevallier N, Rouard H (2018) Subchondral stem cell therapy versus contralateral total knee arthroplasty for osteoarthritis following secondary osteonecrosis of the knee. Int Orthop 42:2563–2571
61. Hommel H, Abdel MP, Perka C (2017) Kinematic femoral alignment with gap balancing and patient-specific instrumentation in total knee arthroplasty: a randomized clinical trial. Eur J Orthop Surg Traumatol 27:683–688
62. Hsieh CJ, DeJong G, Vita M, Zeymo A, Desale S (2020) Effect of Outpatient Rehabilitation on Functional Mobility After Single Total Knee Arthroplasty: A Randomized Clinical Trial. JAMA Netw Open 3:e2016571
63. Hsu RW-W, Hsu W-H, Shen W-J, Hsu W-B, Chang S-H (2019) Comparison of computer-assisted navigation and conventional instrumentation for bilateral total knee arthroplasty: The outcomes at mid-term follow-up. Medicine (Baltimore) 98:e18083

64. Husby VS, Foss OA, Husby OS, Winther SB (2018) Randomized controlled trial of maximal strength training vs. standard rehabilitation following total knee arthroplasty. Eur J Phys Rehabil Med 54:371–379
65. Jahic D, Omerovic D, Tanovic AT, Dzankovic F, Campara MT (2018) The Effect of Prehabilitation on Postoperative Outcome in Patients Following Primary Total Knee Arthroplasty. Med Arch 72:439–443
66. Jang SW, Kim MS, Koh IJ, Sohn S, Kim C, In Y (2019) Comparison of Anterior-Stabilized and Posterior-Stabilized Total Knee Arthroplasty in the Same Patients: A Prospective Randomized Study. J Arthroplasty 34:1682–1689
67. Jansen MP, Besselink NJ, van Heerwaarden RJ, Custers RJH, Emans PJ, Spruijt S, et al. (2021) Knee Joint Distraction Compared with High Tibial Osteotomy and Total Knee Arthroplasty: Two-Year Clinical, Radiographic, and Biochemical Marker Outcomes of Two Randomized Controlled Trials. Cartilage 12:181–191
68. Karaborklu Argut S, Celik D, Kilicoglu OI (2021) The Combination of Exercise and Manual Therapy Versus Exercise Alone in Total Knee Arthroplasty Rehabilitation: A Randomized Controlled Clinical Trial. PM R 13:1069–1078
69. Kim B, Lohman E, Yim J (2021) Acupuncture-like Transcutaneous Electrical Nerve Stimulation for Pain, Function, and Biochemical Inflammation After Total Knee Arthroplasty. Altern Ther Health Med 27:28–34
70. Kim J-K, Park IW, Ro DH, Mun B-S, Han H-S, Lee MC (2021) Is a Titanium Implant for Total Knee Arthroplasty Better? A Randomized Controlled Study. J Arthroplasty 36:1302–1309
71. Kim MS, Koh IJ, Kim CK, Choi KY, Jeon JH, In Y (2021) Comparison of Joint Perception Between Posterior-Stabilized and Ultracongruent Total Knee Arthroplasty in the Same Patient. J Bone Joint Surg Am 103:44–52
72. Kim Y-H, Park J-W, Jang Y-S (2021) Long-Term (Up to 27 Years) Prospective, Randomized Study of Mobile-Bearing and Fixed-Bearing Total Knee Arthroplasties in Patients <60 Years of Age With Osteoarthritis. J Arthroplasty 36:1330–1335
73. Kim Y-H, Park J-W, Kim J-S (2018) 2017 Chitranjan S. Ranawat Award: Does Computer Navigation in Knee Arthroplasty Improve Functional Outcomes in Young Patients? A Randomized Study. Clin Orthop Relat Res 476:6–15
74. Kim Y-H, Park J-W, Kim J-S (2018) Comparison of High-Flexion Fixed-Bearing and High-Flexion Mobile-Bearing Total Knee Arthroplasties-A Prospective Randomized Study. J Arthroplasty 33:130–135
75. Kim Y-H, Park J-W, Kim J-S (2017) The Clinical Outcome of Computer-Navigated Compared with Conventional Knee Arthroplasty in the Same Patients: A Prospective, Randomized, Double-Blind, Long-Term Study. J Bone Joint Surg Am 99:989–996
76. Klika AK, Yakubek G, Piuzzi N, Calabrese G, Barsoum WK, Higuera CA (2022) Neuromuscular Electrical Stimulation Use after Total Knee Arthroplasty Improves Early Return to Function: A Randomized Trial. J Knee Surg 35:104–111
77. Kline PW, Melanson EL, Sullivan WJ, Blatchford PJ, Miller MJ, Stevens-Lapsley JE, et al. (2019) Improving Physical Activity Through Adjunct Telerehabilitation Following Total Knee Arthroplasty: Randomized Controlled Trial Protocol. Phys Ther 99:37–45
78. Knifsund J, Niinimaki T, Nurmi H, Toom A, Keemu H, Laaksonen I, et al. (2021) Functional results of total-knee arthroplasty versus medial unicompartmental arthroplasty:

two-year results of a randomised, assessor-blinded multicentre trial. BMJ Open 11:e046731
79. Koh IJ, Kim MS, Sohn S, Song KY, Choi NY, In Y (2019) Patients undergoing total knee arthroplasty using a contemporary patella-friendly implant are unaware of any differences due to patellar resurfacing. Knee Surg Sports Traumatol Arthrosc 27:1156–1164
80. Komaris D-S, Govind C, Murphy AJ, Clarke J, Ewen A, Leonard H, et al. (2021) Implant design affects walking and stair navigation after total knee arthroplasty: a double-blinded randomised controlled trial. J Orthop Surg Res 16:177
81. Koppens D, Rytter S, Munk S, Dalsgaard J, Sørensen OG, Hansen TB, et al. (2019) Equal tibial component fixation of a mobile-bearing and fixed-bearing medial unicompartmental knee arthroplasty: a randomized controlled RSA study with 2-year follow-up. Acta Orthop 90:575–581
82. Kosse NM, Heesterbeek PJC, Schimmel JJP, van Hellemondt GG, Wymenga AB, Defoort KC (2018) Stability and alignment do not improve by using patient-specific instrumentation in total knee arthroplasty: a randomized controlled trial. Knee Surg Sports Traumatol Arthrosc 26:1792–1799
83. Kulshrestha V, Sood M, Kanade S, Kumar S, Datta B, Mittal G (2020) Early Outcomes of Medial Pivot Total Knee Arthroplasty Compared to Posterior-Stabilized Design: A Randomized Controlled Trial. Clin Orthop Surg 12:178–186
84. Lachiewicz PF, O'Dell JA (2019) Prospective randomized trial of standard versus highly crosslinked tibial polyethylene in primary posterior-stabilized total knee arthroplasty: clinical and radiological follow-up at 2 to 11 years. Bone Joint J 101-B:33–39
85. Laende EK, Richardson CG, Dunbar MJ (2019) A randomized controlled trial of tibial component migration with kinematic alignment using patient-specific instrumentation versus mechanical alignment using computer-assisted surgery in total knee arthroplasty. Bone Joint J 101-B:929–940
86. Lee H-G, An J, Lee B-H (2021) The Effect of Progressive Dynamic Balance Training on Physical Function, The Ability to Balance and Quality of Life Among Elderly Women Who Underwent a Total Knee Arthroplasty: A Double-Blind Randomized Control Trial. Int J Environ Res Public Health 18(5), 2513
87. Lee J-Y, Kim J-H, Lee B-H (2020) Effect of Dynamic Balance Exercises Based on Visual Feedback on Physical Function, Balance Ability, and Depression in Women after Bilateral Total Knee Arthroplasty: A Randomized Controlled Trial. Int J Environ Res Public Health 17(9), 3203
88. Lee O-S, Lee YS (2018) Effect of the Referencing System on the Posterior Condylar Offset and Anterior Flange-Bone Contact in Posterior Cruciate-Substituting Total Knee Arthroplasty. J Arthroplasty 33:1069–1075
89. Leenders AM, Kort NP, Koenraadt KLM, van Geenen RCI, Most J, Kerens B, et al. (2022) Patient-specific instruments do not show advantage over conventional instruments in unicompartmental knee arthroplasty at 2 year follow-up: a prospective, two-centre, randomised, double-blind, controlled trial. Knee Surg Sports Traumatol Arthrosc 30:918–927
90. Lenguerrand E, Artz N, Marques E, Sanderson E, Lewis K, Murray J, et al. (2020) Effect of Group-Based Outpatient Physical Therapy on Function After Total Knee Replacement: Results From a Multicenter Randomized Controlled Trial. Arthritis Care Res (Hoboken) 72:768–777

91. Lenguerrand E, Beswick AD, Whitehouse MR, Wylde V, Blom AW (2018) Outcomes following hip and knee replacement in diabetic versus nondiabetic patients and well versus poorly controlled diabetic patients: a prospective cohort study. Acta orthopaedica 89(4), 399-405
92. Li L, Cheng S, Wang G, Duan G, Zhang Y (2019) Tai chi chuan exercises improve functional outcomes and quality of life in patients with primary total knee arthroplasty due to knee osteoarthritis. Complement Ther Clin Pract 35:121–125
93. Li X, Qi X-B, Han X, Wang W, Liu J-N, Guo J-C, et al. (2017) Effects of sealing the intramedullary femoral canal in total knee arthroplasty: A randomized study. Medicine (Baltimore) 96:e7388
94. Li Y, Wang XG, Tian H, Tao LY (2021) Effect of femoral component rotational alignment in total knee arthroplasty by using 3D-printed patient-specific instruments: a prospective randomized control study. Zhonghua Yi Xue Za Zhi 101:2766–2771
95. Li Z, Cheng W, Sun L, Yao Y, Cao Q, Ye S, et al. (2018) Mini-subvastus versus medial parapatellar approach for total knee arthroplasty: a prospective randomized controlled study. Int Orthop 42:543–549
96. Liao C-D, Tsauo J-Y, Chiu Y-S, Ku J-W, Huang S-W, Liou T-H (2020) Effects of Elastic Resistance Exercise After Total Knee Replacement on Muscle Mass and Physical Function in Elderly Women With Osteoarthritis: A Randomized Controlled Trial. Am J Phys Med Rehabil 99:381–389
97. Lijia T, Rui-Xi Z, Long G, Lei C, Yujie L, Jin-Yun H, et al. (2017) Relationship between patients in hospital affects recovery from total knee arthroplasty (TKA)-A prospective study. J Orthop Sci 22:880–885
98. Liljensøe A, Laursen JO, Bliddal H, Søballe K, Mechlenburg I (2021) Weight Loss Intervention Before Total Knee Replacement: A 12-Month Randomized Controlled Trial. Scand J Surg 110:3–12
99. Liow MHL, Goh GS-H, Wong MK, Chin PL, Tay DK-J, Yeo S-J (2017) Robotic-assisted total knee arthroplasty may lead to improvement in quality-of-life measures: a 2-year follow-up of a prospective randomized trial. Knee Surg Sports Traumatol Arthrosc 25:2942–2951
100. Louwerens JKG, Hockers N, Achten G, Sierevelt IN, Nolte PA, van Hove RP (2021) No clinical difference between TiN-coated versus uncoated cementless CoCrMo mobile-bearing total knee arthroplasty; 10-year follow-up of a randomized controlled trial. Knee Surg Sports Traumatol Arthrosc 29:750–756
101. Loyd BJ, Jennings JM, Judd DL, Kim RH, Wolfe P, Dennis DA, et al. (2017) Influence of Hip Abductor Strength on Functional Outcomes Before and After Total Knee Arthroplasty: Post Hoc Analysis of a Randomized Controlled Trial. 97(9), 896-903
102. Lützner J, Beyer F, Dexel J, Fritzsche H, Lützner C, Kirschner S (2017) No difference in range of motion between ultracongruent and posterior stabilized design in total knee arthroplasty: a randomized controlled trial. Knee Surg Sports Traumatol Arthrosc 25:3515–3521
103. MacDessi SJ, Bhimani A, Burns AWR, Chen DB, Leong AKL, Molnar RB, et al. (2019) Does soft tissue balancing using intraoperative pressure sensors improve clinical outcomes in total knee arthroplasty? A protocol of a multicentre randomised controlled trial. BMJ Open 9:e027812

104. McEwen PJ, Dlaska CE, Jovanovic IA, Doma K, Brandon BJ (2020) Computer-Assisted Kinematic and Mechanical Axis Total Knee Arthroplasty: A Prospective Randomized Controlled Trial of Bilateral Simultaneous Surgery. J Arthroplasty 35:443–450
105. Mochizuki T, Yano K, Ikari K, Okazaki K (2021) Difference in patient-reported outcomes of various patellar component designs in total knee arthroplasty: A randomized clinical study. J Orthop Surg (Hong Kong) 29:2309499021996068
106. Moorthy V, Lai MC, Liow MHL, Chen JY, Pang HN, Chia S-L, et al. (2021) Similar postoperative outcomes after total knee arthroplasty with measured resection and gap balancing techniques using a contemporary knee system: a randomized controlled trial. Knee Surg Sports Traumatol Arthrosc 29:3178–3185
107. Mortensen JF, Rasmussen LE, Østgaard SE, Kappel A, Madsen F, Schrøder HM, et al. (2019) Randomized clinical trial of medial unicompartmentel versus total knee arthroplasty for anteromedial tibio-femoral osteoarthritis. The study-protocol. BMC Musculoskelet Disord 20:119
108. Moukarzel M, Guillot A, Di Rienzo F, Hoyek N (2019) The therapeutic role of motor imagery during the chronic phase after total knee arthroplasty: a pilot randomized controlled trial. Eur J Phys Rehabil Med 55:806–815
109. Mushtaq N, Liddle AD, Isaac D, Dillow K, Gill P (2018) Patient-Reported Outcomes following Single- and Multiple-Radius Total Knee Replacement: A Randomized, Controlled Trial. J Knee Surg 31:87–91
110. Nam D, Lawrie CM, Salih R, Nahhas CR, Barrack RL, Nunley RM (2019) Cemented Versus Cementless Total Knee Arthroplasty of the Same Modern Design: A Prospective, Randomized Trial. J Bone Joint Surg Am 101:1185–1192
111. Nguyen C, Boutron I, Roren A, Anract P, Beaudreuil J, Biau D, et al. (2022) Effect of Prehabilitation Before Total Knee Replacement for Knee Osteoarthritis on Functional Outcomes: A Randomized Clinical Trial. JAMA Netw Open 5:e221462
112. Nishitani K, Furu M, Nakamura S, Kuriyama S, Ishikawa M, Ito H, et al. (2018) No differences in patient-reported outcomes between medial pivot insert and symmetrical insert in total knee arthroplasty: A randomized analysis. Knee 25:1254–1261
113. Nivbrant NO, Khan RJK, Fick DP, Haebich S, Smith E (2020) Cementless Versus Cemented Tibial Fixation in Posterior Stabilized Total Knee Replacement: A Randomized Trial. J Bone Joint Surg Am 102:1075–1082
114. Orndahl CM, Perera RA, Riddle DL (2021) Associations Between Physical Therapy Visits and Pain and Physical Function After Knee Arthroplasty: A Cross-Lagged Panel Analysis of People Who Catastrophize About Pain Prior to Surgery. Phys Ther 101
115. Paravlic AH, Maffulli N, Kovač S, Pisot R (2020) Home-based motor imagery intervention improves functional performance following total knee arthroplasty in the short term: a randomized controlled trial. J Orthop Surg Res 15:451
116. Park CH, Kang SG, Bae DK, Song SJ (2019) Mid-term clinical and radiological results do not differ between fixed- and mobile-bearing total knee arthroplasty using titanium-nitride-coated posterior-stabilized prostheses: a prospective randomized controlled trial. Knee Surg Sports Traumatol Arthrosc 27:1165–1173
117. Pasqualotto S, Demey G, Michelet A, Nover L, Saffarini M, Dejour D (2020) Bipolar Sealers Do not Improve Blood Loss or Functional Outcomes of Primary Total Knee Arthroplasty. J Knee Surg 33:62–66

118. Percope de Andrade MA, Moreira de Abreu Silva G, de Oliveira Campos TV, Guen Kasuya Barbosa D, da Silva Leite D, Teodoro Rezende MV, et al. (2022) A new methodology for patient education in total knee arthroplasty: a randomized controlled trial. Eur J Orthop Surg Traumatol 32:107–112
119. Petursson G, Fenstad AM, Gøthesen Ø, Dyrhovden GS, Hallan G, Röhrl SM, et al. (2018) Computer-Assisted Compared with Conventional Total Knee Replacement: A Multicenter Parallel-Group Randomized Controlled Trial. J Bone Joint Surg Am 100:1265–1274
120. Pinsornsak P, Kanitnate S, Boontanapibul K (2021) The effect of immediate post-operative knee range of motion photographs on post-operative range of motion after total knee arthroplasty : An assessor-blinded randomized controlled clinical trial in sixty patients. Int Orthop 45:101–107
121. Piva SR, Almeida GJ, Gil AB, DiGioia AM, Helsel DL, Sowa GA (2017) Effect of Comprehensive Behavioral and Exercise Intervention on Physical Function and Activity Participation After Total Knee Replacement: A Pilot Randomized Study. Arthritis Care Res (Hoboken) 69:1855–1862
122. Piva SR, Schneider MJ, Moore CG, Catelani MB, Gil AB, Klatt BA, et al. (2019) Effectiveness of Later-Stage Exercise Programs vs Usual Medical Care on Physical Function and Activity After Total Knee Replacement: A Randomized Clinical Trial. JAMA Netw Open 2:e190018
123. Postler A, Beyer F, Lützner C, Tille E, Lützner J (2018) Similar outcome during short-term follow-up after coated and uncoated total knee arthroplasty: a randomized controlled study. Knee Surg Sports Traumatol Arthrosc 26:3459–3467
124. Powell AJ, Crua E, Chong BC, Gordon R, McAuslan A, Pitto RP, et al. (2018) A randomized prospective study comparing mobile-bearing against fixed-bearing PFC Sigma cruciate-retaining total knee arthroplasties with ten-year minimum follow-up. Bone Joint J 100-B:1336–1344
125. Pozzi F, White DK, Snyder-Mackler L, Zeni JA (2020) Restoring physical function after knee replacement: a cross sectional comparison of progressive strengthening vs standard physical therapy. Physiother Theory Pract 36:122–133
126. Prats-Uribe A, Kolovos S, Berencsi K, Carr A, Judge A, Silman A, et al. (2021) Unicompartmental compared with total knee replacement for patients with multimorbidities: a cohort study using propensity score stratification and inverse probability weighting. Health Technol Assess 25:1–126
127. Prvu Bettger J, Green CL, Holmes DN, Chokshi A, Mather RC 3rd, Hoch BT, et al. (2020) Effects of Virtual Exercise Rehabilitation In-Home Therapy Compared with Traditional Care After Total Knee Arthroplasty: VERITAS, a Randomized Controlled Trial. J Bone Joint Surg Am 102:101–109
128. Raaij TM van, Meij E van der, Vries AJ de, Raay JJAM van (2021) Patellar Resurfacing Does Not Improve Clinical Outcome in Patients with Symptomatic Tricompartmental Knee Osteoarthritis. An RCT Study of 40 Patients Receiving Primary Cruciate Retaining Total Knee Arthroplasty. J Knee Surg 34:1503–1509
129. Riddle DL, Slover J, Keefe FJ, Ang DC, Dumenci L, Perera RA (2021) Racial Differences in Pain and Function Following Knee Arthroplasty: A Secondary Analysis From a Multicenter Randomized Clinical Trial. Arthritis Care Res (Hoboken) 73:810–817

130. Roig-Casasús S, Blasco JM, López-Bueno L, Blasco-Igual MC (2018) Balance Training With a Dynamometric Platform Following Total Knee Replacement: A Randomized Controlled Trial. J Geriatr Phys Ther 41:204–209
131. Russo LR, Benedetti MG, Mariani E, Roberti di Sarsina T, Zaffagnini S (2017) The Videoinsight(®) Method: improving early results following total knee arthroplasty. Knee Surg Sports Traumatol Arthrosc 25:2967–2971
132. Sano Y, Iwata A, Wanaka H, Matsui M, Yamamoto S, Koyanagi J, et al. (2018) An easy and safe training method for trunk function improves mobility in total knee arthroplasty patients: A quasi-randomized controlled trial. PLoS One 13:e0204884
133. Sappey-Marinier E, de Abreu FGA, O'Loughlin P, Gaillard R, Neyret P, Lustig S, et al. (2020) No difference in patellar position between mobile-bearing and fixed-bearing total knee arthroplasty for medial osteoarthritis: a prospective randomized study. Knee Surg Sports Traumatol Arthrosc 28:1542–1550
134. Sappey-Marinier E, Swan J, Maucort-Boulch D, Batailler C, Malatray M, Neyret P, et al. (2022) No significant clinical and radiological differences between fixed versus mobile bearing total knee replacement using the same semi-constrained implant type: a randomized controlled trial with mean 10 years follow-up. Knee Surg Sports Traumatol Arthrosc 30:603–611
135. Sattler LN, Hing WA, Vertullo CJ (2019) Pedaling-Based Protocol Superior to a 10-Exercise, Non-Pedaling Protocol for Postoperative Rehabilitation After Total Knee Replacement: A Randomized Controlled Trial. J Bone Joint Surg Am 101:688–695
136. Şavkin R, Büker N, Güngör HR (2021) The effects of preoperative neuromuscular electrical stimulation on the postoperative quadriceps muscle strength and functional status in patients with fast-track total knee arthroplasty. Acta Orthop Belg 87:735–744
137. Scarvell JM, Perriman DM, Smith PN, Campbell DG, Bruce WJM, Nivbrant B (2017) Total Knee Arthroplasty Using Bicruciate-Stabilized or Posterior-Stabilized Knee Implants Provided Comparable Outcomes at 2 Years: A Prospective, Multicenter, Randomized, Controlled, Clinical Trial of Patient Outcomes. J Arthroplasty 32:3356-3363.e1
138. Schache MB, McClelland JA, Webster KE (2019) Incorporating hip abductor strengthening exercises into a rehabilitation program did not improve outcomes in people following total knee arthroplasty: a randomised trial. J Physiother 65:136–143
139. Schotanus MGM, Boonen B, van der Weegen W, Hoekstra H, van Drumpt R, Borghans R, et al. (2019) No difference in mid-term survival and clinical outcome between patient-specific and conventional instrumented total knee arthroplasty: a randomized controlled trial. Knee Surg Sports Traumatol Arthrosc 27:1463–1468
140. Schotanus MGM, Pilot P, Kaptein BL, Draijer WF, Tilman PBJ, Vos R, et al. (2017) No difference in terms of radiostereometric analysis between fixed- and mobile-bearing total knee arthroplasty: a randomized, single-blind, controlled trial. Knee Surg Sports Traumatol Arthrosc 25:2978–2985
141. Schotanus MGM, Pilot P, Vos R, Kort NP (2017) No difference in joint awareness after mobile- and fixed-bearing total knee arthroplasty: 3-year follow-up of a randomized controlled trial. Eur J Orthop Surg Traumatol 27:1151–1155
142. Schrednitzki D, Beier A, Marx A, Halder AM (2020) No Major Functional Benefit After Bicompartmental Knee Arthroplasty Compared to Total Knee Arthroplasty at 5-Year Follow-Up. J Arthroplasty 35:3587–3593

143. Schulz M, Krohne B, Röder W, Sander K (2018) Randomized, prospective, monocentric study to compare the outcome of continuous passive motion and controlled active motion after total knee arthroplasty. Technol Health Care 26:499–506
144. Scott DF (2018) Prospective Randomized Comparison of Posterior-Stabilized Versus Condylar-Stabilized Total Knee Arthroplasty: Final Report of a Five-Year Study. J Arthroplasty 33:1384–1388
145. Sershon RA, Fricka KB, Hamilton WG, Nam D, Parks NL, DeBenedetti A, et al. (2022) Early Results of a Randomized Controlled Trial of Partial Versus Total Knee Arthroplasty. J Arthroplasty 37:S94–S97
146. Sindhupakorn B, Numpaisal P-O, Thienpratharn S, Jomkoh D (2019) A home visit program versus a non-home visit program in total knee replacement patients: a randomized controlled trial. J Orthop Surg Res 14:405
147. Skoffer B, Maribo T, Mechlenburg I, Korsgaard CG, Søballe K, Dalgas U (2020) Efficacy of preoperative progressive resistance training in patients undergoing total knee arthroplasty: 12-month follow-up data from a randomized controlled trial. Clin Rehabil 34:82–90
148. Smith TO, Parsons S, Ooms A, Dutton S, Fordham B, Garrett A, et al. (2022) Randomised controlled trial of a behaviour change physiotherapy intervention to increase physical activity following hip and knee replacement: the PEP-TALK trial. BMJ Open 12:e061373
149. Smith WA, Zucker-Levin A, Mihalko WM, Williams M, Loftin M, Gurney JG (2019) A Randomized Study of Exercise and Fitness Trackers in Obese Patients After Total Knee Arthroplasty. Orthop Clin North Am 50:35–45
150. Stolarczyk A, Nagraba L, Mitek T, Stolarczyk M, Deszczyński JM, Jakucinski M (2018) Does Patient-Specific Instrumentation Improve Femoral and Tibial Component Alignment in Total Knee Arthroplasty? A Prospective Randomized Study. Adv Exp Med Biol 1096:11–17
151. Sun J-N, Chen W, Zhang Y, Zhang Y, Feng S, Chen X-Y (2020) Does cognitive behavioral education reduce pain and improve joint function in patients after total knee arthroplasty? A randomized controlled trial. Int Orthop 44:2027–2035
152. Sun M-L, Zhang Y, Peng Y, Fu D-J, Fan H-Q, He R (2020) Accuracy of a Novel 3D-Printed Patient-Specific Intramedullary Guide to Control Femoral Component Rotation in Total Knee Arthroplasty. Orthop Surg 12:429–441
153. Tammachote N, Panichkul P, Kanitnate S (2018) Comparison of Customized Cutting Block and Conventional Cutting Instrument in Total Knee Arthroplasty: A Randomized Controlled Trial. J Arthroplasty 33:746-751.e3
154. Tapasvi SR, Shekhar A, Patil SS, Dipane MV, Chowdhry M, McPherson EJ (2020) Comparison of Gap Balancing vs Measured Resection Technique in Patients Undergoing Simultaneous Bilateral Total Knee Arthroplasty: One Technique per Knee. J Arthroplasty 35:732–740
155. Teissier V, Leclercq R, Schiano-Lomoriello S, Nizard R, Portier H (2020) Does eccentric-concentric resistance training improve early functional outcomes compared to concentric resistance training after total knee arthroplasty? Gait Posture 79:145–151
156. Thiengwittayaporn S, Srungboonmee K, Chiamtrakool B (2019) Resurfacing in a Posterior-Stabilized Total Knee Arthroplasty Reduces Patellar Crepitus Complication: A Randomized, Controlled Trial. J Arthroplasty 34:1969–1974

157. Thiengwittayaporn S, Sumranwanich N, Hongku N, Sansawat P (2021) Onlay Patellar Resurfacing in a Posterior-Stabilized Total Knee Arthroplasty Increases Patellar Crepitus Complication: A Randomized, Controlled Trial. J Arthroplasty 36:3443–3450
158. Thiengwittayaporn S, Tangtrakul P, Hongku N, Tunyasuwanakul R (2021) Patellar Denervation Reduces Postoperative Anterior Knee Pain After Patellar Resurfacing Total Knee Arthroplasty: A Randomized Controlled Trial. J Arthroplasty 36:1295–1301
159. Thijs E, Theeuwen D, Boonen B, van Haaren E, Hendrickx R, Vos R, et al. (2020) Comparable clinical outcome and implant longevity after CT- or MRI-based patient-specific instruments for total knee arthroplasty: a 2-year follow-up of a RCT. Knee Surg Sports Traumatol Arthrosc 28:1821–1826
160. Tsubosaka M, Kamenaga T, Kuroda Y, Takayama K, Hashimoto S, Ishida K, et al. (2021) Accelerometer-Based Portable Navigation System Is Useful for Tibial Bone Cutting in Modified Kinematically Aligned Total Knee Arthroplasty. J Knee Surg 34:870–876
161. Tsukada Y, Matsuse H, Shinozaki N, Takano Y, Nago T, Shiba N (2020) Combined Application of Electrically Stimulated Antagonist Muscle Contraction and Volitional Muscle Contraction Prevents Muscle Strength Weakness and Promotes Physical Function Recovery After Total Knee Arthroplasty: A Randomized Controlled Trial. Kurume Med J 65:145–154
162. Turgeon TR, Cameron B, Burnell CD, Hedden DR, Bohm ER (2019) A double-blind randomized controlled trial of total knee replacement using patient-specific cutting block instrumentation versus standard instrumentation. Can J Surg 62:460–467
163. Ugbeye ME, Itakpe SE, Ayodabo OJ (2018) Subvastus versus Medial Parapatellar Approach in Primary Total Knee Replacement: An Assessment of Early Function. West Afr J Med 35:15–19
164. Van Hamersveld KT, Marang-Van De Mheen PJ, Nelissen RGHH, Toksvig-Larsen S (2018) Migration of all-polyethylene compared with metal-backed tibial components in cemented total knee arthroplasty. Acta Orthopaedica 89:412–417
165. Van Hamersveld KT, Marang-Van De Mheen PJ, Van Der Heide HJL, Van Der Linden-Van Der Zwaag HMJ, Valstar ER, Nelissen RGHH (2018) Migration and clinical outcome of mobile-bearing versus fixed-bearing single-radius total knee arthroplasty. Acta Orthop 89:190-196
166. Van Leeuwen JAMJ, Snorrason F, Röhrl SM (2018) No radiological and clinical advantages with patient-specific positioning guides in total knee replacement. Acta Orthop 89:89–94
167. Verra WC, van Hilten JA, Honohan Á, van Zwet EW, van der Bom JG, Nelissen RGHH (2018) The effect of a fibrin sealant on knee function after total knee replacement surgery. Results from the FIRST trial. A multicenter randomized controlled trial. PLoS One 13:e0200804
168. Wang Z, Ji Y, Bao H, Hou J, Cheng Y-X (2021) Patellar retraction versus eversion on functional outcomes in total knee replacement: a randomized controlled study protocol. J Orthop Surg Res 16:381
169. Wellman SS, Klement MR, Queen RM (2017) Performance Comparison of Single-Radius Versus Multiple-Curve Femoral Component in Total Knee Arthroplasty: A Prospective, Randomized Study Using the Lower Quarter Y-Balance Test. Orthopedics 40:e1074–e1080

170. Wirries N, Ezechieli M, Stimpel K, Skutek M (2020) Impact of continuous passive motion on rehabilitation following total knee arthroplasty. Physiother Res Int 25:e1869
171. Wood TJ, Winemaker MJ, Williams DS, Petruccelli DT, Tushinski DM, de Beer J de V (2021) Randomized Controlled Trial of Sensor-Guided Knee Balancing Compared to Standard Balancing Technique in Total Knee Arthroplasty. J Arthroplasty 36:953–957
172. Wu L-P, Mayr HO, Zhang X, Huang Y-Q, Chen Y-Z, Li Y-M (2022) Knee Scores of Patients with Non-Lateral Compartmental Knee Osteoarthritis Undergoing Mobile, Fixed-Bearing Unicompartmental Knee and Total Knee Arthroplasties: A Randomized Controlled Trial. Orthop Surg 14:73–87
173. Xu J, Zhang J, Wang X-Q, Wang X-L, Wu Y, Chen C-C, et al. (2017) Effect of joint mobilization techniques for primary total knee arthroplasty: Study protocol for a randomized controlled trial. Medicine (Baltimore) 96:e8827
174. Xu T, Yang D, Liu K, Gao Q, Lu H, Qiao Y, et al. (2021) Efficacy and safety of a self-developed home-based enhanced knee flexion exercise program compared with standard supervised physiotherapy to improve mobility and quality of life after total knee arthroplasty: a randomized control study. J Orthop Surg Res 16:382
175. Yuan D, Zhang Q-S, Zhang K, Cao Y-W, Chen G-H, Ling Z-Z, et al. (2020) Total Knee Arthroplasty Using a Medial Pivot or Posterior Cruciate-Stabilizing Prosthesis in Chinese Patients. J Knee Surg 33:892–898
176. Yuan F, Sun Z, Wang H, Chen Y, Yu J (2019) Clinical and radiologic outcomes of two patellar resection techniques during total knee arthroplasty: a prospective randomized controlled study. Int Orthop 43:2293–2301
177. Yuan M, Wang Y, Wang H, Ding Z, Xiao Q, Zhou Z (2020) Optimal Handling of the Patella in Tourniquet-Free Total Knee Arthroplasty: Eversion or Lateral Retraction? Orthop Surg 12:1870–1881
178. Zan P, Fan L, Liu K, Yang Y, Hu S, Li G (2017) Reduction Osteotomy versus Extensive Release on Clinical Outcome Measures in Simultaneous Bilateral Total Knee Arthroplasty. Med Sci Monit 23:3817–3823
179. Zhang Y, Zhang Y, Sun J-N, An L, Chen X-Y, Feng S (2021) Comparison of outcomes between gap balancing and measured resection techniques for total knee arthroplasty: A prospective, randomized, controlled trial. Acta Orthop Traumatol Turc 55:239–245
180. Zhu M, Chen JY, Chong HC, Pang HN, Tay DKJ, Chia S-L, et al. (2018) No Difference in Functional Outcomes after Total Knee Arthroplasty with or without Pinless Navigation. J Knee Surg 31:649–653
181. Zora H, Güngör HR, Bayrak G, Şavkın R, Büker N (2020) Does mini-midvastus approach have an advantageous effect on rapid recovery protocols over medial parapatellar approach in total knee arthroplasty? Jt Dis Relat Surg 31:571–581
